# Polyphenol-Driven Interfacial Control: How *Achillea millefolium* Extract Modulates Mild Carbon Steel Corrosion in Acid and Neutral Media

**DOI:** 10.3390/ma19102008

**Published:** 2026-05-12

**Authors:** Gabriela Elena Badea, Ioana Maior, Anda Ioana Grațiela Petrehele, Oana Delia Stănășel, Alexandrina Fodor, Mioara Sebeșan, Simona Dzitac, Camelia Daniela Ionaș

**Affiliations:** 1 Department of Chemistry, Faculty of Informatics and Sciences, University of Oradea, 1 Universitatii Str., 410087 Oradea, Romania; gbadea@uoradea.ro (G.E.B.); ostanasel@uoradea.ro (O.D.S.); msebesan@uoradea.ro (M.S.);; 2 Department of Inorganic Chemistry, Physical Chemistry and Electrochemistry, Faculty of Chemical Engineering and Biotechnology, National University of Science and Technology Politehnica Bucharest, 313 Splaiul Independentei, 060042 Bucharest, Romania; 3 Department of Energy Engineering, Faculty of Energy Engineering and Industrial Management, University of Oradea, 1 Universitatii Str., 410087 Oradea, Romania; simona.dzitac@gmail.com

**Keywords:** *Achillea millefolium*, hydroethanolic extract, polyphenols, mechanism of corrosion inhibition, mild steel, acid and neutral chloride media

## Abstract

Plant-derived corrosion inhibitors are increasingly investigated due to their rich content of adsorption-active phytochemicals. Four extracts obtained from *Achillea millefolium* were biochemically characterized through spectrophotometric and chromatographic analyses, confirming a substantial polyphenolic content and associated antioxidant capacity. In addition, the hydroethanolic extract (1:1) was examined for its ability to inhibit the corrosion of S235 mild steel in 1 M HCl and neutral medium of 3.5% NaCl by gravimetry, potentiodynamic polarization, open-circuit potential, and electrochemical impedance spectroscopy methods, suggesting that its antioxidant molecules may contribute to the passivation of the metal surface, but in different mechanistic ways. The inhibitory efficiency determined by both the gravimetric method and the Taffel polarization curve method reaches values of 78.53% in HCl 1 M and of 79.65% in NaCl 3.5%, thus demonstrating the contribution of polyphenols from the *Achillea millefolium* extracts to the inhibition of corrosion.

## 1. Introduction

Corrosion remains a pervasive, costly problem across industries: global estimates suggest that the annual cost of corrosion represents several percent of world’s gross domestic product, a substantial avoidable expense if better mitigation were implemented [1,2]. In many industrial operations—notably acid pickling, descaling, industrial cleaning, and oil-well acidizing—mild steel is exposed to strong acids, such as hydrochloric acid. In these environments, the metal undergoes rapid electrochemical dissolution and hydrogen evolution, which accelerate material loss and can also promote hydrogen-related damage (e.g., embrittlement) if not controlled [1,3,4]. Historically effective inhibitors include inorganic passivators (e.g., chromates) and synthetic organic compounds; however, many of these agents are toxic, persistent, or otherwise environmentally problematic, and regulatory frameworks increasingly restrict their use. The carcinogenicity and environmental mobility of hexavalent chromium and similar species are primary drivers for industry to seek safer alternatives [1,2].

Stricter environmental legislation, corporate sustainability commitments, and lifecycle cost considerations now favor solutions that reduce hazardous waste, lower operator exposure, and simplify effluent treatment [2,4,5,6,7]. Replacing or reducing hazardous inhibitors can yield both compliance and long-term cost benefits when inhibitor performance and supply chain scalability are acceptable [2,3,8,9].

Over the past two decades, the proliferation of studies—now numbering in the thousands—on plant extracts has consistently demonstrated their potential as corrosion inhibitors, with plant selection often guided by availability, spontaneity, and the valorization of agricultural, food industry [10], or pharmaceutical byproducts [2,3,11,12]. Despite this extensive activity, the field remains in its early stages due to the vast diversity of botanical species, and this work does not aim to provide an exhaustive view but rather to highlight the inhibitory potential of plant extracts by presenting a set of representative examples from recent literature [11,13,14,15]. Among the recent studies, the following could be mentioned: investigations of various plant extracts as green inhibitors and reviews summarizing extraction, design and applications of polyphenols as inhibitors [11,13,14,16]. These examples illustrate that, even in highly aggressive acidic environments, natural extracts can rival synthetic inhibitors while offering the additional advantages of biodegradability and minimal toxicity [2,4,14].

The rich variety of bioactive constituents found in plants makes their extracts attractive candidates for environmentally friendly corrosion inhibitors. Numerous investigations have shown that medicinal plant extracts can effectively protect mild steel in aggressive environments, as compounds such as alkaloids, flavonoids, tannins, and terpenoids are capable of adsorbing onto the metal surface and mitigating corrosion processes [3,11,17]. The consistently high inhibition efficiencies reported—often exceeding 80–90% in selected systems—further underscore the remarkable potential of these biomolecules [14,16,18,19].

*Achillea millefolium* L., or yarrow, is a well-known member of the Asteraceae family and is one of the best-known species of the genus Achillea, present in folk pharmacopoeias around the world [20,21,22,23,24,25]. The species is a hardy perennial herb that tolerates a variety of habitats, diverse soil types and climatic conditions [21,23,26].

Yarrow has a long, pan-Eurasian and North American history of use for a broad spectrum of ailments: wound care and hemostasis, gastrointestinal complaints, menstrual and uterine disorders, respiratory conditions, and general anti-inflammatory and antispasmodic effects [20,22,27].

Industrial applications beyond medicine demonstrate prior non-pharmaceutical uses that support technological feasibility: textile dyeing and antibacterial finishing, cosmetic ingredients, and phytoremediation/heavy metal tolerance or accumulation—indicating extract stability, processability, and environmental utility [28,29,30,31,32,33,34,35,36]. *Achillea millefolium* extracts and fractions have been investigated for a range of cosmetic and personal care uses driven by their antioxidant, anti-inflammatory and skin-soothing properties; formulations exploit alcohol soluble fractions and essential oils as natural antioxidants and calming agents in creams, masks, and topical preparations, where stability and mildness are required [25,28,37]. These same antioxidant fractions have been trialed as natural preservatives to slow lipid oxidation in food and cosmetic matrices, offering an alternative to synthetic antioxidants [25,28].

In textile and material finishing, yarrow-derived volatiles and phenolic extracts have been tested as antimicrobial and odor control finishes, and as natural functional additives that can impart UV-scavenging or antioxidant functionality to fabrics [34,35]. Such applications use relatively simple extraction and finishing steps, and they demonstrate the plant’s processability for industrial wet processing lines. The dual antimicrobial/antioxidant profile also supports potential use in packaging or fiber treatments where biodegradability and low toxicity are priorities [25,34,35].

Environmental and process engineering uses include phytoremediation, biosorption and effluent treatment: both whole biomass and specific extract fractions have been explored for metal binding capacity and for mitigating oxidative or microbial loads in waste streams [26,36]. The presence of polar phenolics and surface-active triterpenoids suggest mechanisms for complexation and adsorption of heavy metals and for stabilizing emulsions or suspensions during treatment processes [11,26]. These attributes make A. millefolium a candidate for low-cost, low-impact remediation strategies and for the valorization of agricultural residues [7,36].

Finally, several materials engineering and industrial chemistry applications have been proposed or piloted: essential oils and hydrophobic terpenoids as fragrance or solvent components in green formulations; phenolic-rich fractions as antioxidant additives in polymeric materials to retard oxidative degradation; and multifunctional extracts that can act as biobased corrosion inhibitors or coating additives by adsorbing to metal surfaces and forming protective films [11,17,25]. These multifunctional uses—combining adsorption, radical scavenging and hydrophobic barrier effects—provide a direct technological bridge from phytochemistry to corrosion mitigation strategies in acidic environments [11,17].

The performance of green corrosion inhibitors is typically assessed through a combination of electrochemical and analytical methods. Common evaluations include open-circuit potential (OCP), quasi-steady state (potentiodynamic) polarization, electrochemical impedance spectroscopy (EIS), and the classical gravimetric mass-loss technique [1,3,4]. The polarization curve methods, the calculation of the Tafel slope, and EIS are applied to assess the degree of corrosion protection achieved on carbon steel in various acidic or neutral media [3,4,14]. A solution of 1 M HCl is a common laboratory proxy for these aggressive service conditions and for screening inhibitors intended for pickling/descaling applications [14,16,18]. Important contributions to establishing the inhibition mechanisms are made by both analytical methods for identifying biochemical compounds [15,19,25,28,30,37,38,39,40], such as the Folin–Ciocalteu method [36], DPPH, HPLC, GC-MS [36], computational methods (DFT, molecular simulations) [17,28,29,38,41,42], and surface analysis methods: SEM, AFM, XRD [13,14,18,43].

Despite the growing literature on plant extracts as corrosion inhibitors, comparative performance depends strongly on the extraction solvent, plant part, and test conditions. Targeted studies on specific extracts (for example, alcoholic extracts of *Achillea millefolium* against mild steel in 1 M HCl) remain limited [11,17,42]. This gap justifies a focused investigation combining electrochemical testing, surface analysis, and phytochemical profiling to quantify inhibition efficiency and propose a mechanistic model.

This study addresses the existing knowledge gap concerning the limited data on alcoholic extracts of *Achillea millefolium* as corrosion inhibitors for mild steel in 1 M HCl and NaCl 3.5% and therefore aims to quantify their inhibition efficiency through combined electrochemical and surface analyses and, ultimately, by proposing a plausible inhibition mechanism based phytochemical analyses. The alcoholic extract of *Achillea millefolium* likely inhibits mild steel corrosion in acid through a combination of surface adsorption and protective film formation, proven by antioxidant (radical scavenging) activity and metal complexation. These mechanisms can be probed and distinguished using electrochemical and analytical techniques.

## 2. Materials and Methods

### 2.1. Materials

Analytical-grade ethanol, methanol, and sodium carbonate were employed throughout all assays to ensure experimental consistency. Reference antioxidants—including 2,2-diphenyl-1-picrylhydrazyl (DPPH), gallic acid, quercetin, caffeic acid, catechin, sinapic acid and kaempferol—were sourced from Sigma-Aldrich (St. Louis, MO, USA), while the Folin–Ciocalteu reagent was obtained from Merck (Darmstadt, Germany). One of the *Achillea millefolium* extracts included in the analysis was a commercial extract (www.daciaplant.ro, accessed on 9 March 2026).

The dried aerial parts were purchased commercially, finely cut, and subjected to phytochemical extraction. Maceration was performed using a 1:1 ethanol–water mixture, by soaking 10 g of plant powder in 100 mL at 25 °C for 48 h, and vacuum-filtered using a 0.45 µm Millipore (Burlington, MA, USA) membrane. A second extraction method involved refluxing the material in a Soxhlet apparatus: 30 g of dried, finely cut plant were placed in a cellulose cartridge and extracted with 300 mL ethanol for approximately 3 h, during which eight solvent recirculation cycles occurred.

The inhibition efficiency was assessed using solutions prepared by introducing various volumes of the AM extract into 50 mL of 1 M HCl and 3.5%NaCl. These additions corresponded to polyphenol concentrations—expressed as gallic acid equivalents—of 50, 100, 200, and 300 ppm GAE (mg GAE/L) [15,19]. The AM extract was dispensed into the corrosion cell using an LLG automatic microliter pipette, and the same volumetric ratios were applied in the gravimetric tests.

A commercially available S235 mild carbon steel was selected for the experiments. Its elemental composition (wt.%) is C 0.22, Si 0.05, Mn 0.60, Ni 0.30, S 0.04, P 0.04, Cr 0.30, N 0.012, and Cu 0.30, with the remainder being Fe. The alloy has a density of 7.85 g/cm^3^.

For electrochemical measurements, steel plates with an exposed area of 1 cm^2^ were used as working electrodes. Prior to testing, the exposed surfaces were polished sequentially with P600, P2000, and P2500 abrasive papers to obtain a uniform finish, followed by rinsing with distilled water and acetone.

### 2.2. Analytical Methods

#### 2.2.1. Folin–Ciocalteu Method—Determination of Total Phenolic Content

The total phenolic content of each extract was evaluated using the Folin–Ciocalteu method. For the Folin–Ciocalteu assay, 1.7 mL of distilled water, 0.1 mL of either the standard working solution or the sample solution, and 0.2 mL of the Folin–Ciocalteu reagent (diluted 1:10) were placed into a test tube. After brief mixing, the mixture was allowed to stand for one minute, followed by the addition of 1 mL of a 20% Na_2_CO_3_ solution. The reaction mixture was then kept in the dark at room temperature for 90 min. The absorbance of the resulting blue complex was recorded at 765 nm using a SPECORD 210 PLUS UV–Vis spectrophotometer (Analytik-Jena, Jena, Germany). Blanks were prepared in an identical manner, replacing the standard solution with distilled water and the sample solution with the extraction solvent.

Quantification was performed using a calibration curve constructed for gallic acid in the concentration range of 0.1–0.5 mg/mL. Total phenolic content was expressed as mg gallic acid equivalents (GAE) per L of extract. Each measurement was carried out in six replicates, and the reported values represent the corresponding averages.

#### 2.2.2. DPPH Scavenging Method

The hydrogen-donating capacity and radical-scavenging activity of the extract solutions were evaluated using the stable DPPH radical. Each sample was analyzed through a modified version of the DPPH assay. The DPPH reagent was prepared by dissolving 2 mg of DPPH in 50 mL of methanol, yielding a solution of approximately 3 mM. A volume of 0.2 mL of each extract was combined with 2.8 mL of the DPPH solution, and the mixture was incubated in the dark at room temperature. After 30 min, the absorbance was recorded at 517 nm. Gallic acid served as the positive control, while the blank consisted of 2.8 mL methanol mixed with 0.2 mL of the extraction solvent.

The radical-scavenging efficiency of each extract, expressed as DPPH inhibition (%), was calculated using the following equation:

(1) $$DPPH\;inhibition\;\left(\%\right)=\frac{A_{0}-A_{t}}{A_{0}}\cdot 100$$

In this equation, *A*_0_ represents the absorbance of the DPPH control solution (without extract), while A_t_ corresponds to the absorbance measured for the sample containing the extract.

For data interpretation, a calibration curve was constructed using gallic acid standards in the concentration range of 0.1–0.3 mg/mL. These standards were prepared by diluting a 1 mg/mL gallic acid stock solution with distilled water. The same analytical procedure was applied to all extract samples when determining their DPPH radical scavenging activity. *Achillea millefolium* extracts initially prepared at 10 mg/mL were further diluted with the extraction solvent to obtain solutions between 0.1 and 1 mg/mL, and the scavenging capacity was evaluated for each concentration.

#### 2.2.3. RP-HPLC Method

The quantification of phenolic compounds by RP-HPLC was carried out using a Younglin ACME 3000 HPLC system (Young Lin Instrument Co., Ltd., Anyang, Gyeonggi-do, Republic of Korea) equipped with an SP 930D pump module(Shanghai Sunny Hengping Scientific Instrument Co., Ltd., Shanghai, China) and a UV 730D detector (Shanghai Sunny Hengping Scientific Instrument Co., Ltd., Shanghai, China). Analyses were performed under isocratic conditions with a mobile phase consisting of methanol, water, and acetic acid in a 300:700:2 ratio, delivered at a flow rate of 1 mL/min. Separation of the sample constituents was achieved on a reverse-phase YMC-Pack ODS-AQ column (150 mm × 4.6 mm). A 0.2 µL aliquot of each extract was injected at room temperature. Polyphenol quantification in the *Achillea millefolium* extracts was performed using the external standard method.

### 2.3. Electrochemical Methods

Electrochemical experiments were conducted using a Voltalab 40 potentiostat/galvanostat (Radiometer Analytical, Villeurbanne, France), operated through the VoltaMaster 4.0 software for data acquisition and analysis. Measurements were performed in a thermostated electrochemical cell employing a conventional three electrode configuration: an S235 carbon steel electrode (1 cm^2^ exposed area) as the working electrode, a platinum gauze electrode (5 cm^2^ active area) as the counter electrode, and a saturated Ag/AgCl (SSCE) electrode as the reference.

Prior to each experiment, the working electrode surface was mechanically polished with successive grades of emery paper (600–2500), rinsed with distilled water, and dried. The open-circuit potential (OCP) was monitored for 30 min in the acidic medium, both in the absence and presence of *Achillea millefolium* extract, to ensure stabilization before electrochemical testing. Tafel polarization curves were recorded at a scan rate of 0.33 mV/s, beginning from the cathodic region and progressing toward anodic potentials. Potentiodynamic polarization data were processed with potentiostat dedicated software, which automatically performed Tafel fitting and calculated the corresponding kinetic parameters.

For each inhibitor concentration, a minimum of three independent measurements was performed, and the most reproducible dataset from each series was selected for interpretation.

The inhibition efficiency was determined from Tafel plots using the Formula (2):

(2) $$IE=\left(1-\frac{i_{cor}}{i_{cor}^{0}}\right)\times 100,\;\%$$

where i^0^_cor_ and icor (µA/cm^2^) are corrosion current density values in a 1 M HCl solution, without and with AM extract.

Electrochemical impedance spectroscopy (EIS) was performed at the open-circuit potential over a frequency range of 100 kHz to 50 mHz, using an AC perturbation of 10 mV in amplitude. The corrosion inhibition efficiency (IE) was derived from the polarization resistance (R_p_) according to Equation (3):

(3) $$IE,\%=\left(1-\frac{R_{p}^{0}}{R_{p}}\right)\times 100$$

In this expression, R_p_^0^ and R_p_ (Ω·cm^2^) denote the polarization resistance values measured in 1 M HCl in the absence and presence of the AM extract, respectively.

To evaluate the influence of inhibitor concentration, S235 carbon steel electrodes were immersed in 1 M HCl and 3.5% NaCl solutions containing 0 ppm (blank), 50, 100, 200, and 300 ppm GAE of the AM extract.

### 2.4. Gravimetric Method

For the gravimetric analysis, the polished steel coupons were weighed before and after immersion using a Kern ABT 220-5DNM analytical balance (Kern & Sohn GmbH, Balingen, Germany). Each specimen was exposed to 2 mL of test solution for periods of 48, 168, and 216 h. All experiments were carried out at a controlled temperature of 25 ± 1 °C. At the end of each exposure interval, the S235 steel samples were removed, and corrosion products were eliminated through standard chemical cleaning procedures. The coupons were then rinsed with deionized water, followed by an ethanol/acetone mixture, dried, and reweighed to determine the mass loss. Five parallel specimens were used for each experimental condition. The corrosion rate, expressed as the penetration index P (mm/year), was calculated from the measured mass loss according to the following relation:

(4) $$p=\frac{∆m\cdot 8.76}{S\cdot t\cdot \rho }$$

In this equation, Δm represents the mass loss (g), S is the exposed surface area (m^2^), t denotes the immersion time (h), ρ is the density of the carbon steel (g/cm^3^), and 8.76 is the conversion factor used to harmonize the units of time and surface area for expressing the penetration rate.

## 3. Results and Discussion

### 3.1. Biochemical Investigations of Achillea millefolium Extracts

To elucidate the qualitative and quantitative profile of antioxidant constituents, particularly polyphenolic compounds, the extracts were subjected to a complementary set of analytical investigations, including Folin–Ciocalteu assessment, DPPH radical-scavenging evaluation, and HPLC separation.

#### 3.1.1. Folin–Ciocalteu Method

The Folin–Ciocalteu assay is one of the most widely applied methods for quantifying the total polyphenolic content of natural extracts [15,19,41].

The reduced form of the Folin–Ciocalteu reagent exhibits a characteristic absorbance in the 690–750 nm range, allowing its detection by UV–Vis spectrophotometry. In accordance with ISO 14502-1, gallic acid was selected as the reference standard. A calibration curve was established using this compound gallic acid. given its well-documented antioxidant activity and the concentration of phenolic compounds with antioxidant properties is expressed as gallic acid equivalents (mg GAE/L = 1 ppm GAE). Within the 0.05–0.5 mg/mL concentration interval for gallic acid, a linear relationship between absorbance and concentration was obtained. The resulting calibration equation was y = 3.2295x + 0.0657, with a correlation coefficient of R^2^ = 0.9984, indicating excellent linearity.

Four types of extracts from *Achillea millefolium* were comparatively analyzed as follows: hydroalcoholic extract Dacia Plant (AM-DP); Soxhlet ethanolic extract from dried aerial parts of plants (AM-SOX) prepared in the laboratory; and two hydroalcoholic extracts from dried plants prepared in the laboratory, one from the plant’s aerial parts (AM-L) and other from yarrow’s flowering tops (AM-F).

The comparative assessment of the four extracts revealed clear differences in total polyphenol content, driven both by solvent polarity and by the plant organ subjected to extraction. The extracts obtained from aerial parts showed marked variability across the three solvent systems (AM-S-100% ethanolic, AM-L and AM-F 50:50 hydroethanolic, and AM-DP 30:70 hydroethanolic), confirming the well-established sensitivity of phenolic compounds to changes in the ethanol–water ratio. In general, increasing the proportion of water enhanced the solubilization of several phenolic subclasses, which was reflected in the higher values observed for the hydroethanolic extracts compared with the absolute ethanolic extract.

**Figure 1:**
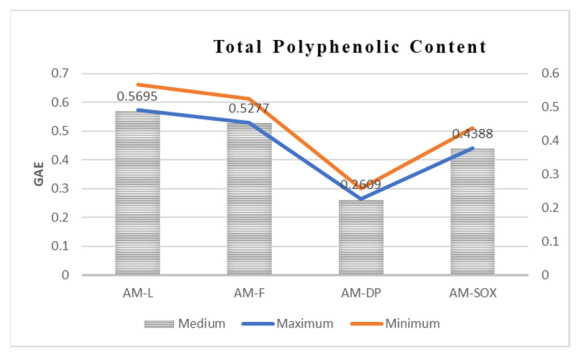
Comparative diagram of total polyphenolic content for *Achillea millefolium* extract solutions.

In Figure 1, it can be seen that the highest concentration of polyphenols, expressed in grams equivalent of gallic acid, was obtained for the hydroalcoholic extract of *Achillea millefolium*, namely from the aerial parts of the plant (AM-L). The difference between the polyphenols found in the AM-L extract from the aerial parts and the one from the inflorescence, AM-F, is insignificant: 0.5695 mg/mL for AM-L and 0.5227 mg/mL for AM-F. A slightly lower concentration of the total amount of polyphenols was found for the reflux extraction. This decrease of the polyphenol’s concentration is explained by the thermal degradation of some components, which can give a positive redox reaction with the Folin–Ciocalteu reagent. A significantly lower concentration of polyphenols, only 0.2609 mg/mL, was obtained for the extract of *Achillea millefolium* from Dacia Plant (AM-DP) for which the period between the preparation of the extract and the determination of the total amount of polyphenols is significantly longer than in the case of the other *Achillea millefolium* extracts, freshly prepared in the laboratory (AM-L, AM-F and AM-SOX).

A comparison with the commercial Dacia Plant product (30:70 hydroethanolic extract) provided an external benchmark for evaluating the performance of the experimental extracts. The laboratory-prepared 50:50 extract exhibited polyphenol levels comparable to or exceeding those of the commercial formulation, suggesting that the extraction methodology employed is both efficient and reproducible. At the same time, the differences observed between the commercial product and the experimental extracts may reflect variations in raw material quality, processing conditions, or industrial standardization practices.

Overall, the results demonstrate that solvent polarity and plant part are key determinants of polyphenol extraction efficiency, and that benchmarking against a commercial product enhances the applied relevance of the findings. These insights support the targeted optimization of extraction protocols, according to the desired phenolic profile. The commercial Dacia Plant extract (AM-DP) was used for electrochemical investigations.

#### 3.1.2. DPPH Method

The health-promoting properties of plant extracts are largely attributed to the strong antioxidant activity associated with their polyphenolic constituents. To evaluate the antioxidant potential of the *Achillea millefolium* extracts, their free-radical-scavenging capacity was assessed. The solution exhibits a deep purple color, characterized by a maximum absorbance at 517 nm. After a 30 min reaction time, the absorbance was measured, and the antioxidant activity of the four investigated extracts was determined (Table 1).

**Figure 2:**
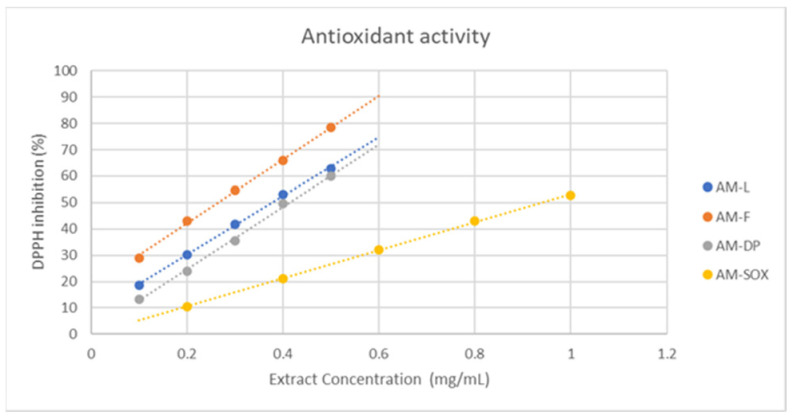
Variation in the antioxidant activities of *Achillea millefolium* extracts using the DPPH method.

Graphs of the percentage inhibition variation of DPPH as a function of concentration were plotted for each series of concentrations, for standard solutions of gallic acid and also for all yarrow extracts studied. The linear variation equation and the correlation coefficient, R2, were determined for each sample (Figure 2).

For both gallic acid standards and *Achillea millefolium* extracts, the concentration required to inhibit 50% of the DPPH activity (IC_50_) was determined.

**Figure 3:**
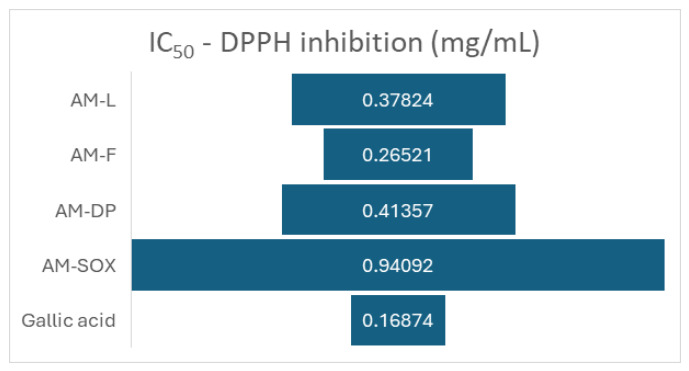
Extracts concentrations of *Achillea millefolium* samples required to inhibit 50% of DPPH.

Figure 3 shows that all the analyzed samples have a weaker antioxidant activity than that the gallic acid solution (IC50 0.16874 mg/mL). In the case of the *Achillea millefolium* extracts taken in this study, the highest measured antioxidant activity against DPPH was presented by the hydroalcoholic extract from the flowers (AM-F), prepared at room temperature, where IC50 was 0.26521 mg/mL. In comparison, for the ethanol–water 1:1 extract from aerial parts of the plant (AM-L), a decrease in antioxidant activity was observed: IC50 was found to be 0.37824 mg/mL. The extract AM-DP prepared by Dacia Plant and also from the aerial parts of the plant, displayed an even weaker antioxidant activity (IC50 0.41357 mg/mL). The worst antioxidant activity, significantly lower than that of the other samples analyzed, was recorded in the case of *Achillea millefolium* aerial parts extracted in boiling ethanol in a Soxhlet device, indicating an alteration of antioxidant activity upon heating.

#### 3.1.3. Analysis of Phenols by RP-HPLC Method

**Figure 4:**
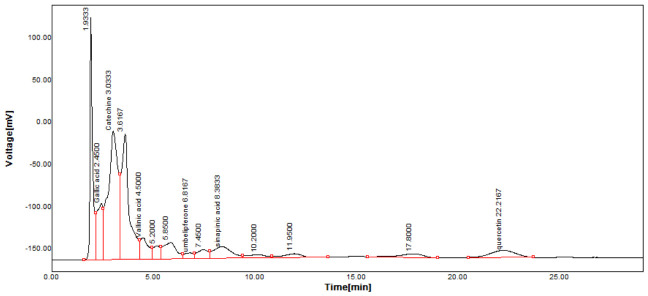
HPLC chromatogram of *Achillea millefolium* extract AM-L.

A reverse-phase HPLC analysis was employed to quantify selected polyphenolic constituents present in the prepared extracts. As a preliminary step, calibration curves for the targeted polyphenols were established using standard solutions. Calibration curves were plotted for standard solutions diluted with methanol, with concentrations between 0.01 and 0.10 mg/mL for gallic acid and catechin and between 0.1 and 0.3 mg/mL for caffeic acid, sinapic acid, kaempferol and quercetin, using the regression linear method. The calibration curves obtained with analytical standards and the correlation coefficient are shown in Table 2. Figure 4 presents the chromatographic profile obtained for the *Achillea millefolium* extract.

As shown in Table 3, the AM-L extract—freshly prepared in the laboratory using a hydroalcoholic solvent—contained the highest concentration of gallic acid. In contrast, the AM-DP extract exhibited the greatest levels of caffeic acid and sinapic acid. The concentrations of catechin in both AM-L and AM-DP extracts were very close. With the exception of the kaempferol concentration, which was almost the same in all three samples, and that of gallic acid, which was quite high in AM-SOX, the concentrations of the other determined antioxidants were significantly lower in AM-SOX in comparation with the extracts obtained by maceration in the 1:1 ethanol–water mixture at room temperature. These findings indicate that several polyphenolic constituents undergo thermal degradation in the AM-SOX extract obtained by ethanol reflux, a trend consistent with the results from both total polyphenol quantification and antioxidant activity measurements. The HPLC analysis further refines this interpretation by clearly identifying which specific polyphenolic compounds are most susceptible to heat-induced degradation.

All analyzed samples contained the following polyphenolic constituents: gallic acid; the flavanols catechin, kaempferol, and quercetin; and caffeic and sinapic acids. The concentrations of these compounds varied among extracts, depending on both the extraction technique and the solvent employed.

Taken together, the Folin–Ciocalteu assay, the DPPH radical scavenging test, and the RP HPLC profiling converge toward the same outcome: all *Achillea millefolium* extracts contain quantifiable and chemically meaningful levels of polyphenols and related antioxidant constituents. The spectrophotometric data confirmed substantial total phenolic content, particularly in AM L (0.5695 mg GAE/mL) and AM F (0.5277 mg GAE/mL), while DPPH measurements demonstrated measurable radical scavenging capacity across all samples, with AM F showing the lowest IC_50_ (0.26521 mg/mL). A HPLC analysis further validated the presence of structurally diverse phenolics—gallic acid, catechin, caffeic and sinapic acids, rutin, isoquercitrin, and luteolin—compounds whose π electron systems and redox-active functional groups are well documented in the literature as potential anodic, cathodic, or mixed-type corrosion inhibitors. The coherence of these three analytical methods thus substantiates the extract’s richness in polyphenols capable of participating in adsorption, electron-transfer modulation, and surface passivation processes relevant to corrosion inhibition.

### 3.2. Corrosion Studies in the Presence of Achillea millefolium Extract

To assess the performance of various natural plant extracts as corrosion inhibitors for steel in acidic media and to identify the optimal concentration of the AM extract, several electrochemical and gravimetric techniques were employed. The investigation included open-circuit potential (OCP), Tafel polarization, electrochemical impedance spectroscopy (EIS), and mass-loss measurements.

The interval of 50–300 ppm GAE for inhibitor was selected because the 300 ppm level already provided inhibition efficiencies close to 80%, consistent with typical performance reported for green inhibitors. In addition, the polyphenol content of the extracts showed natural variability (≈440 mg GAE/L on average, down to ~260 mg GAE/L), making 300 ppm a realistic lower-limit concentration attainable from plant-derived materials.

#### 3.2.1. Gravimetric Experiments

Gravimetric measurements were performed on S235 mild steel specimens in 1 M HCl and in solution of 3.5% NaCl for exposure periods of 48, 168, and 216 h at 25 °C. Corrosion rates, penetration indices, p (mm/year), and inhibition efficiencies were calculated for each inhibitor concentration (Table 4 and Table 5).

In the acidic medium, the penetration indices exhibit a systematic decrease with increasing inhibitor concentration, confirming the progressive development of a stable protective film on the steel surface. At 48 h, for instance, the penetration rate decreases from 7.6862 mm·year^−1^ in the uninhibited solution to 1.8267 mm·year^−1^ at 300 ppm, and similar trends are observed at 168 and 216 h, where the values reach 0.9894 and 0.4643 mm·year^−1^, respectively. These results indicate a consistent reduction in uniform corrosion, supported by inhibition efficiencies exceeding 70% at higher concentrations, suggesting favorable adsorption and the formation of a relatively compact surface layer in the acidic environment.

In the neutral 3.5% NaCl solution, the penetration indices are naturally much lower than in HCl, reflecting the intrinsically reduced uniform corrosion rate. However, the variations in p are more irregular, and the decreases are less pronounced, which is characteristic of systems where localized corrosion mechanisms dominate. At 216 h, for example, p decreases only from 0.0290 to 0.0151 mm/year at 300 ppm, and the corresponding inhibition efficiencies remain modest, ranging from 8.42% to 48.09%, depending on concentration. This behavior indicates that the organic film formed in the neutral chloride medium is less stable and more susceptible to local breakdown, resulting in a lower overall protective effect.

From the mechanistic interpretation, the marked differences between the two media can be rationalized by considering the adsorption behavior of the inhibitor and the role of chloride ions. In acidic solutions, the surface of mild steel is strongly protonated, and the phenolic and flavonoid constituents of the extract are more readily protonated as well, which enhances electrostatic interactions and promotes the formation of a uniform inhibitor layer. Under these conditions, corrosion proceeds predominantly through uniform dissolution, allowing the inhibitor to effectively reduce the active surface area.

Due to their abundance of hydroxyl (–OH) and carbonyl (–C=O) functional groups, polyphenolic constituents exhibit a strong propensity to adsorb onto steel surfaces and to promote the formation of protective interfacial films. Several studies have shown that plant extracts rich in polyphenols interact readily with iron, generating a thin yet mechanically resilient passive layer. The adsorption of phenolic and flavonoid molecules is generally attributed to the ability of –OH and –C=O groups to establish coordination interactions or hydrogen bonding with surface Fe sites, thereby hindering the access of aggressive ions and stabilizing the nascent protective film.

In contrast, in the neutral chloride-containing medium, Cl^−^ ions compete directly with the inhibitor molecules for adsorption sites on the metal surface. Owing to their small size and high mobility, chloride ions can easily penetrate the organic film, destabilizing it and generating localized anodic sites that initiate pitting. This localized attack leads to progressive perforation of the protective layer, which explains both the lower inhibition efficiencies and their variability over time. Furthermore, the accumulation of chloride ions within pit cavities promotes local acidification, accelerating metal dissolution and further limiting the inhibitor’s ability to control the process.

**Figure 5:**
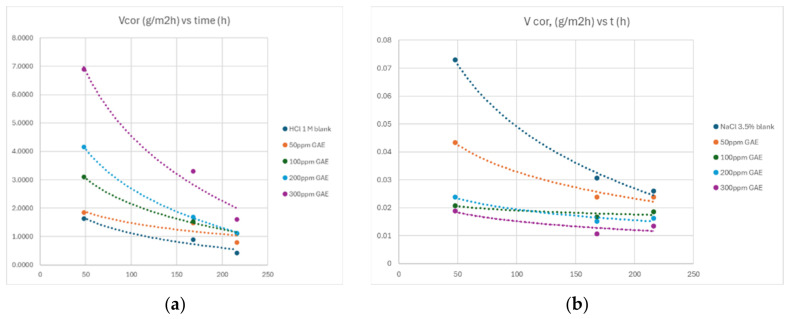
Time-resolved comparison of corrosion behavior in 1 M HCl (**a**) and 3.5% NaCl (**b**) solutions with different polyphenol amounts of the AM extract.

The comparative evolution of the corrosion rate in acidic and neutral environments (Figure 5) provides a consistent basis for interpreting the gravimetric data discussed above. In 1 M HCl containing various concentrations of polyphenols, the corrosion rate of mild steel is initially high but decreases sharply as exposure progresses, following a logarithmic decay that gradually stabilizes toward a quasi-steady value.

This behavior reflects the progressive reduction of the active metallic surface as corrosion products and inhibitor molecules accumulate, forming a partially protective layer that slows down the dissolution process. The absolute values fall within the typical range reported for acidic environments (5–15 g·m^−2^·h^−1^), confirming that the system is governed by uniform corrosion, where the inhibitor can effectively adsorb and reduce the exposed surface area.

The logarithmic decrease in corrosion rate observed in both media further supports the proposed mechanism. In acidic solution, the decline is dominated by the gradual stabilization of an inhibitor-rich surface layer, whereas in the neutral chloride medium, the decrease reflects the partial accumulation of corrosion products that temporarily hinder dissolution, even though the surface remains vulnerable to pitting. The convergence toward a relatively constant value in both cases indicates the establishment of a dynamic balance between metal dissolution and surface film formation or degradation.

Overall, the graphical trends corroborate the mechanistic interpretation derived from the gravimetric measurements: the inhibitor performs significantly better in the acidic medium due to favorable adsorption and the predominance of uniform corrosion, while its efficiency is inherently limited in the neutral chloride environment by the competitive and destabilizing action of Cl^−^ ions.

#### 3.2.2. Potentiodynamic Polarization Experiments

**Figure 6:**
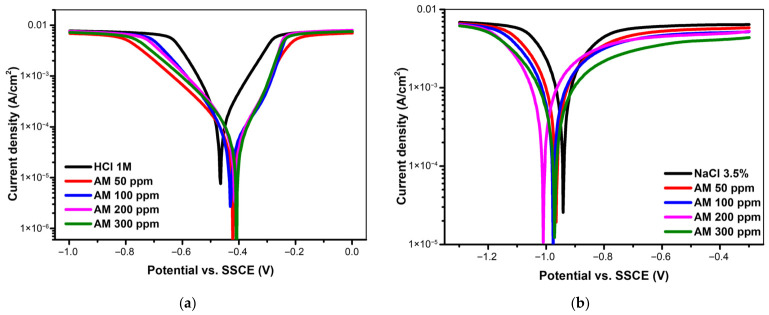
Tafel polarization curves of carbon steel in (**a**) acidic and (**b**) neutral media in the presence and absence of the AM extract.

Figure 6a,b illustrate the potentiodynamic polarization curves (Tafel plots) for carbon steel exposed to 1 M HCl and 3.5% NaCl solutions, respectively, each containing the AM inhibitor at concentrations ranging from 50 to 300 ppm at room temperature. As shown in Figure 6a, increasing the inhibitor concentration shifts the polarization curves toward more negative corrosion potentials (E_corr_), indicating a pronounced suppression of the cathodic reaction. Nevertheless, according to the criteria applied in this study (Table 6), the potential displacement remains below 85 mV, confirming that the AM extract behaves as a mixed-type inhibitor. Additionally, both the anodic (b*_a_*) and cathodic (b*_c_*) Tafel slopes exhibit noticeable changes in the presence of the inhibitor.

The corrosion parameters obtained from the Tafel plots—calculated by linear extrapolation—are summarized in Table 6 and Table 7. These include the corrosion potential (E_corr_, mV), corrosion current density (i_corr_, mA/cm^2^), polarization resistance (R*_p_*, Ω·cm^2^), anodic and cathodic Tafel slopes (b*_a_* and b*_c_*, mV/dec), corrosion rate (P, mm/year), and inhibition efficiency (IE, %).

Another notable observation is that increasing the AM concentration progressively shifts the polarization curves in Figure 6 toward lower corrosion current densities. As shown in Table 6, the corrosion current density (i_corr_) of carbon steel in the uninhibited 1 M HCl solution is relatively high, approximately 0.15 mA/cm^2^ at an E_corr_ of −460.9 mV. The addition of only 50 ppm AM markedly decreases i_corr_ to 0.0787 mA/cm^2^ at −417.1 mV, indicating a substantial improvement in corrosion resistance. Further increases in inhibitor concentration—100 ppm and above—continue to reduce the corrosion current density, reaching a minimum value of about 0.0322 mA/cm^2^ at −411.03 mV when 300 ppm AM is used.

With respect to the inhibition efficiency of the AM extract, the calculated IE values increase sharply from 50 ppm onward, reaching a maximum at 300 ppm, where an efficiency of approximately 78.53% is achieved.

In the neutral medium (3.5% NaCl), the absence of the inhibitor (Figure 6b) results in a simultaneous shift of both anodic and cathodic branches toward higher current densities, reflecting accelerated iron dissolution and an intensified oxygen-reduction process. Introducing the AM extract markedly suppresses the current density in both domains, indicating that the inhibitor interferes with both anodic metal oxidation and cathodic reduction pathways. This concurrent attenuation confirms the mixed-type character of the AM extract, consistent with the formation of an adsorbed organic layer that restricts charge transfer at the steel surface [43,44].

The inhibitory effect becomes increasingly pronounced at 300 ppm, where the corrosion current density (i_corr_) exhibits a substantial decline relative to the uninhibited solution. The anodic branch shows a reduced slope, suggesting that AM effectively limits the active dissolution of Fe^2+^ by preferential adsorption at anodic sites. Simultaneously, the cathodic branch displays diminished slopes, indicating that the inhibitor also impedes oxygen-reduction kinetics, likely through partial blockage of cathodic regions by adsorbed organic species. This dual-site adsorption mechanism aligns with behaviors reported for other plant-derived inhibitors, where the combined action of polar functional groups and hydrophobic chains suppresses both anodic and cathodic reactions, thereby reducing the overall corrosion rate.

Table 7 shows that the corrosion current density (i_corr_) of carbon steel in the uninhibited 1 M HCl solution is relatively high, approximately 0.15 mA/cm^2^ at an E_corr_ of −460.9 mV. Introducing a small amount of AM extract (50 ppm) produces a marked decrease in i_corr_, lowering it to 0.0787 mA/cm^2^ at −417.1 mV, which reflects a substantial improvement in corrosion resistance. Increasing the inhibitor concentration to 100 ppm and beyond results in a progressive decline in the corrosion current density, reaching its minimum value—around 0.0322 mA/cm^2^ at −411.03 mV—when 300 ppm of AM is added. This monotonic decrease demonstrates that the inhibitory action strengthens with concentration, consistent with enhanced surface coverage and more effective blocking of active corrosion sites.

The inhibition efficiency (IE, %) calculated for the AM extract shows a pronounced concentration-dependent increase. Starting from 50 ppm, the IE values rise sharply and continue to improve with increasing inhibitor dosage, reaching a maximum at 300 ppm, where an efficiency of approximately 78.53% is obtained. This trend reflects the progressive enhancement of surface coverage and the more effective blocking of active corrosion sites as the concentration of AM increases.

Chloride ions act as activators of the anodic reaction, causing the corrosion potential to shift towards more negative values and increasing the corrosion rate. In their presence, the mechanism of the metal dissolution reaction changes, involving the formation of adsorbed intermediate species (MCl)_ads_, which can prevent the appearance of passive layers. At high pH values, the degree of adsorption of Cl^−^ ions on the metal surface is low, due to competition with OH^−^ ions. If the pH decreases, the adsorption of OH^–^ is lower and consequently the adsorption of Cl^−^ ions increase, which intensifies the corrosion rate. The presence of Cl^−^ ions in solution intensifies the corrosion of active metals, making the formation of the passive film more difficult, as well as promoting the corrosion of passive metals, by inducing pitting corrosion. This pH-dependent effect refers to chloride-containing systems in which the corrosion process is governed by the competitive adsorption of Cl^−^ and OH^−^; it does not apply to the direct comparison between strongly acidic media (1 M HCl) and neutral NaCl solutions, which follow fundamentally different corrosion mechanisms.

The inhibition efficiencies obtained from polarization measurements were consistently higher than those derived from weight-loss experiments, both in acidic and neutral media. This behavior is well documented for organic inhibitors and can be rationalized by considering the intrinsic differences between the two techniques. Polarization curves provide an instantaneous electrochemical snapshot of the system, typically recorded within a few minutes, during which the inhibitor film is still compact and relatively undisturbed. Under these short-term conditions, the adsorbed polyphenolic species from *Achillea millefolium* interact strongly with the steel surface through the coordination of –OH and –C=O groups to surface Fe sites, leading to an apparently high degree of surface coverage and, consequently, higher inhibition efficiencies.

In contrast, gravimetric measurements integrate the corrosion process over extended exposure times, during which the initially formed film may undergo gradual reorganization, thinning, or partial desorption. Local defects, micro-heterogeneities, and the progressive penetration of aggressive ions contribute to a more realistic assessment of long-term protection. As a result, the inhibition efficiencies obtained gravimetrically in an acidic medium (30.41–74.14%) are slightly lower than those from polarization (47.53–78.53%), reflecting the cumulative impact of film degradation that is not captured by short-term electrochemical measurements.

The discrepancy becomes significantly more pronounced in a neutral chloride-containing solution. While polarization still indicates relatively high efficiencies (49.08–79.65%), the gravimetric values drop substantially (8.42–48.09%). This divergence suggests that, although the inhibitor film appears effective at early stages, its stability is markedly compromised during prolonged immersion. In neutral media, corrosion is strongly influenced by oxygen reduction and by the competitive adsorption of chloride ions, which progressively disrupt the protective layer and promote a localized attack. Such processes are not fully reflected in the instantaneous polarization response but are clearly manifested in the cumulative mass loss. The large difference between the two methods therefore indicates that the inhibitor film formed in neutral solution is less resilient and more susceptible to chloride-induced breakdown over time.

The combined electrochemical and gravimetric results highlight that *Achillea millefolium* extract forms a relatively stable protective film in an acidic medium, whereas in a neutral chloride solution, the film is only transiently effective, with long-term stability significantly reduced by competitive adsorption and localized corrosion processes.

#### 3.2.3. OCP Experiments

**Figure 7:**
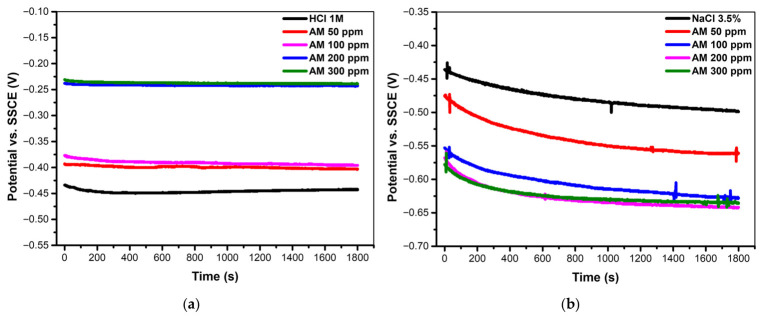
Time-dependent evolution of OCP of mild steel in (**a**) acidic and (**b**) neutral media, with and without the AM inhibitor.

To ensure that a stable electrochemical condition was reached, the open-circuit potential (OCP) was monitored until only minimal fluctuations were observed. The resulting curves are presented in Figure 7a,b. In an acidic medium, the addition of the AM extract produces a marked shift of the potential toward more positive values—typically by 50–200 mV relative to the blank—an effect that increases proportionally with inhibitor concentration. Approximately 200 s after immersion, all curves exhibit a slight displacement toward more negative potential, a behavior commonly attributed to the initial dissolution of the native oxide film. Beyond this transient stage, the OCP stabilizes for all concentrations of AM, suggesting the progressive formation of a protective surface layer. The enhanced stability observed at higher inhibitor concentrations indicates that this film becomes more compact and effective as the amount of AM increases.

In the neutral medium (3.5% NaCl), all OCP curves exhibit a gradual decrease until a stable potential is reached after approximately 25 min (Figure 7). During the first 10 min of immersion, a pronounced shift toward more negative potentials is observed for every tested condition, reflecting the rapid breakdown of the initial surface film and the onset of active corrosion. Increasing the concentration of the AM extract causes the OCP values to shift further in the negative direction, with a maximum difference of about 70–80 mV relative to the blank. Since this displacement remains below the commonly accepted threshold of 85 mV, the AM extract can be classified as a mixed-type inhibitor, influencing both anodic and cathodic processes in the neutral environment.

The OCP results presented in Figure 7 reinforce the mechanistic interpretation established earlier: a stable, strongly adsorbed inhibitor film in acidic medium and a transient, chloride sensitive film in neutral solution, whose degradation over time explains the divergence between electrochemical and gravimetric inhibition efficiencies.

Figure 7 shows the time-dependent evolution of the open-circuit potential for mild steel in the two corrosive media, with and without the *Achillea millefolium* extract. In an acidic solution, the OCP curves exhibit a gradual shift toward slightly more positive values during the initial immersion period, followed by a stable plateau. This behavior indicates the progressive formation of a coherent inhibitor film on the steel surface, consistent with the strong adsorption of polyphenolic constituents through –OH and –C=O functional groups. The stabilization of the potential reflects a surface that becomes progressively less reactive, supporting both the higher inhibition efficiencies obtained from polarization curves and the relatively small discrepancy between electrochemical and gravimetric measurements in this medium.

In contrast, the OCP response in neutral chloride solution (Figure 7b) displays noticeable fluctuations and a tendency to drift toward more negative values over time. Such behavior is characteristic of an unstable or discontinuous protective film, which undergoes progressive degradation under the competitive action of chloride ions. The negative shift of the potential suggests localized breakdown events and the exposure of fresh anodic sites, indicating that the inhibitor film formed in neutral medium lacks long-term stability. This observation aligns with the pronounced difference between the high short-term efficiencies obtained from polarization and the significantly lower long-term efficiencies measured gravimetrically. The OCP behavior therefore provides independent confirmation that, although the inhibitor film appears initially effective in neutral solution, it is progressively compromised during immersion due to chloride-induced destabilization.

#### 3.2.4. EIS Experiments

Electrochemical impedance spectroscopy (EIS) was employed to evaluate the corrosion behavior of carbon steel in 1 M HCl and 3.5% NaCl solutions containing various concentrations of the AM extract.

**Figure 8:**
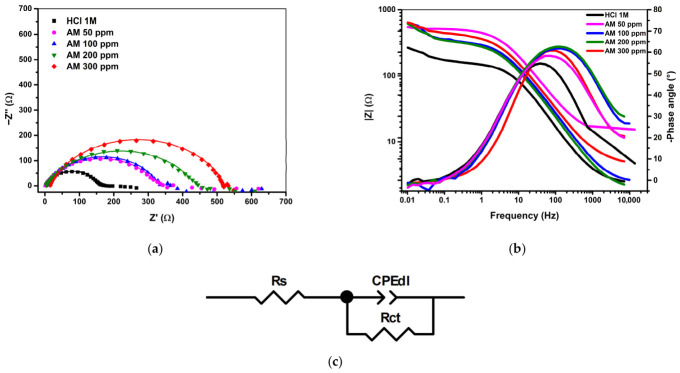
Nyquist (**a**) and Bode (**b**) plots and the equivalent circuit (**c**) of carbon steel in acidic media with increasing AM inhibitor concentrations.

Figure 8 and Table 8 present the electrochemical impedance spectroscopy (EIS) results obtained in this study. The figure includes the Nyquist (a) and Bode (b) plots, together with the equivalent circuit (c) used for fitting. Experimental data are shown as dotted curves, while the continuous lines represent the fitted responses generated using ZView software.

Table 8 summarizes the electrochemical parameters obtained from fitting the EIS data, including the solution resistance, charge-transfer resistance, constant-phase element parameters, and χ^2^ values corresponding to the equivalent circuit shown in Figure 8.

In the acidic medium (1 M HCl), the Nyquist diagrams display a single, moderately depressed capacitive loop characteristic of a charge-transfer-controlled corrosion process (Figure 8). The semicircle diameter, which reflects the interfacial charge-transfer resistance, increases progressively with the addition of the AM extract, indicating a steady improvement in polarization resistance and a corresponding reduction in corrosion rate. This behavior arises from the formation of an adsorbed protective layer that limits chloride-ion penetration and suppresses electron-transfer processes associated with iron dissolution.

At 300 ppm, the impedance response exhibits the largest semicircle, confirming the highest degree of surface coverage and the most effective inhibition. The Bode spectra further support this interpretation, showing a consistent rise in |Z| at low frequencies and a single, well-defined phase-angle maximum, indicative of one dominant relaxation process. The Rs–(Rct‖CPE) equivalent circuit provides an adequate description of the system, and the CPE exponent (n = 0.76–0.80) reflects the expected surface heterogeneity of carbon steel in acidic environments. Overall, the progressive enlargement of the capacitive loops with increasing inhibitor concentration confirms the development of a compact and highly protective film at the metal–solution interface.

Compared with the higher and more monotonic inhibition efficiencies obtained from gravimetric measurements and Tafel polarization in 1 M HCl, the efficiencies derived from EIS (Table 8) are slightly lower and exhibit greater sensitivity to interfacial heterogeneity. Even so, the pronounced increase in charge-transfer resistance and the systematic decrease in double-layer capacitance across all AM concentrations clearly confirm the same protective trend identified by the other techniques. This convergence of results indicates that, although EIS is more stringent in detecting defects or discontinuities within the surface film, it fully supports the conclusion that the AM extract forms a stable, adherent, and highly effective inhibitory layer on carbon steel in acidic medium.

The calculated double-layer capacitance (C_dl_) decreases progressively with increasing AM concentration, indicating either a reduction in the local dielectric constant or an effective increase in the thickness of the electrical double layer. This behavior is consistent with the displacement of interfacial water molecules by adsorbed organic constituents and with the incorporation of long-chain hydrocarbon structures that lower the permittivity near the metal surface. A similar decline in C_dl_ is typically associated with the formation of dense, adherent inhibitor films that block active corrosion sites and limit charge-transfer processes.

**Figure 9:**
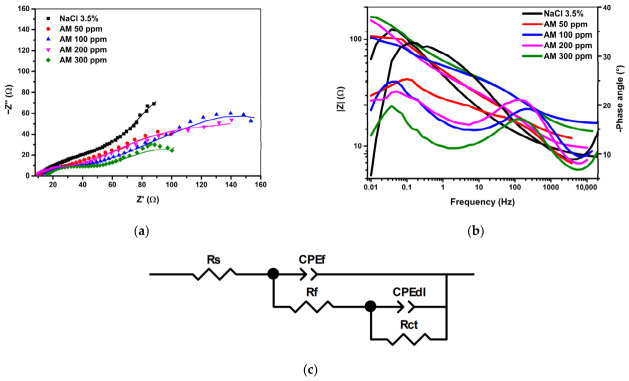
Nyquist (**a**) and Bode (**b**) plots and the equivalent circuit (**c**) of carbon steel in neutral media with increasing AM inhibitor concentrations.

Figure 9 and Table 9 present the EIS results for mild steel in 3.5% NaCl, comparing the uninhibited solution with the AM-treated systems; the figure includes the Nyquist and Bode plots, together with the corresponding two-time-constant equivalent circuit, while the table lists the fitted electrochemical parameters that describe the chloride-induced corrosion process and its modification by the polyphenolic extract.

In the 3.5% NaCl medium, the equivalent circuit consists of the solution resistance (Rs), followed by two parallel R–CPE branches that reflect the presence of two distinct relaxation processes: the high frequency branch (Rct and CPEdl) represents the charge-transfer reaction at the steel/electrolyte interface and the non-ideal double layer capacitance, while the low frequency branch (Rf and CPEf) corresponds to the inhibitor-related interfacial process associated with the formation and evolution of the adsorbed polyphenolic film. Together, these elements capture both the heterogeneous double layer and the slower surface film dynamics characteristic of corrosion in chloride media with plant-derived inhibitors.

The inhibition efficiency (IE) in 3.5% NaCl was calculated from the electrochemical impedance spectroscopy data using the polarization resistance obtained from the fitted equivalent circuit. In accordance with the referenced methodology [43], the total polarization resistance was taken as the sum of the resistive elements in the circuit,

(5) $$R_{p}=R_{ct}+R_{f}$$

in which R_p_ represents the total polarization resistance, defined as the sum of charge-transfer resistance (R_ct_) and the film resistance (R_f_). Then IE% was determined with Equation (3).

Conversely, in neutral chloride solution (Figure 9), the semicircles remain significantly smaller and more depressed, indicating a heterogeneous and chloride-sensitive layer whose protective effect, although improved at higher AM concentrations, remains markedly inferior to that observed in acid.

In the neutral medium (3.5% NaCl), the inhibitor-free solution displays a single depressed semicircle of small diameter in the Nyquist plot. Upon addition of the AM extract, the impedance response evolves into two distinct features. This behavior is typically associated with partial re-dissolution of the passive film on carbon steel or with relaxation processes involving adsorbed inhibitor species. As shown in Figure 9, increasing the AM concentration leads to a marked rise in impedance, evidenced by the enlargement of the suppressed semicircles. This trend indicates that the AM extract enhances corrosion protection by forming surface layers that hinder electron mobility and effectively limit charge-transfer processes at the steel–solution interface.

The decrease in CPE_dl_ with increasing AM concentration reflects an effective increase in double-layer thickness, as the inhibitor molecules adsorb onto the steel surface. Accordingly, the highest CPE_dl_ value and the lowest R*p* value observed in the blank solution indicate severe corrosion, consistent with extensive Cl^−^ adsorption across the steel surface.

The pronounced rise in R*p* combined with the reduction in CPE_dl_ at optimal inhibitor concentration supports a mixed physisorption–chemisorption mechanism. Physisorption is likely driven by long-chain alkanes, whereas chemisorption arises from polar functional groups (alcohols, esters) capable of interacting with Fe surface atoms. This interpretation aligns with reports on natural extracts, where combined physical and chemical adsorption enhances the barrier properties of the protective film.

The lower n values obtained in the NaCl system reflect the higher interfacial heterogeneity characteristic of chloride-induced surface roughening and partial film formation, rather than a deficiency in the fitting procedure.

This impedance behavior reconfirms the trends previously identified through gravimetric measurements, OCP evolution, and Tafel polarization analysis. The stable and coherent film inferred from the positive OCP shift and the high polarization efficiencies in acidic medium is fully supported by the large Rct values obtained from EIS. In contrast, the fluctuating OCP response, the discrepancy between short-term electrochemical efficiencies and long-term gravimetric performance, and the modest increase in Rct in neutral solution collectively demonstrate that the inhibitor film is progressively destabilized by chloride ions. Thus, the EIS results provide a final and independent validation of the mechanistic picture established earlier, highlighting the contrasting stability of the AM-derived protective layer in the two corrosive environments.

Although adsorption isotherms were not determined in the present study, the inhibitory behavior of *Achillea millefolium* extract is consistent with the adsorption-controlled mechanisms, a trend extensively documented for polyphenol-rich botanical extracts. The abundance of hydroxyl (–OH) and carbonyl (–C=O) groups in phenolic and flavonoid constituents enables electron donation toward vacant Fe d-orbitals, promoting chemisorption, while their polar regions support additional hydrogen bonding and physisorption. Numerous studies have shown that such natural inhibitors typically follow Langmuir or, in some cases, Freundlich adsorption isotherms, reflecting the formation of a quasi-monolayer protective film on metal surfaces [11,43]. This trend is well documented in recent reviews, which report that most plant extracts exhibit Langmuir-type adsorption, driven by electron-rich aromatic systems and heteroatoms capable of coordinating with surface Fe atoms, thereby blocking active sites and reducing the accessibility of corrosive ions [34].

**Figure 10:**
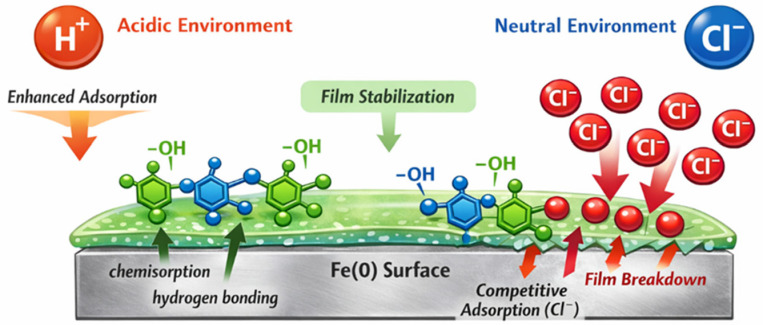
Mechanistic illustration of inhibitor adsorption and film stability in acidic (HCl) and neutral environments (NaCl) of *Achillea millefolium* extract on mild steel.

This mechanistic interpretation is visually summarized in Figure 10, which illustrates the adsorption of polyphenolic constituents onto the steel surface and the differing stability of the resulting protective film in acidic versus neutral environments.

Reports on other plant extracts containing polyphenols similar to those identified in the present *Achillea millefolium* extract have shown, through SEM, AFM, and XRD analyses, that such constituents form protective adsorbed films on metal surfaces, in full agreement with the inhibition mechanisms inferred from electrochemical measurements. Table 10 summarizes several literature reports on polyphenol-containing plant extracts relevant to the inhibition mechanism discussed above.

## 4. Conclusions

The biochemical analyses confirmed that the *Achillea millefolium* extracts contain a consistent pool of polyphenols and antioxidant compounds known to interact effectively with metallic surfaces. The electrochemical and gravimetric results converge toward the same mechanism, showing the formation of a stable, protective film in an acidic medium and a less stable, chloride-sensitive layer in a neutral solution. Overall, the extract behaves as a promising green inhibitor under acidic conditions, while its performance in neutral saline environments is limited by the susceptibility of the adsorbed layer to Cl^−^ attack. Its relevance lies in its natural origin, safety, and renewability rather than in exceptional efficiency.

## Figures and Tables

**Table 1:** Comparative DPPH radical-scavenging activity of the analyzed extracts.

No.	Sample	Type of Extract	Equation	R^2^	IC_50_ (mg/mL)
1	AM-L	ethanol–water 1:1	y = 0.1117x + 7.75	0.9988	0.37824
2	AM-F	ethanol–water 1:1	y = 0.1214x + 17.804	0.9986	0.26521
3	AM-DP	ethanol–water 1:1	y = 0.119x + 0.785	0.9977	0.41357
4	AM-SOX	ethanol Soxhlet	y = 0.0532x − 0.057	0.9996	0.94092
5	Gallic Acid	water	y = 0.2905x + 0.9807	0.9973	0.16874

**Table 2:** Results obtained by RP-HPLC analysis for the *Achillea millefolium* extract.

Compound	Structure	Retention Time (min)	Equation	R^2^
Gallic acid	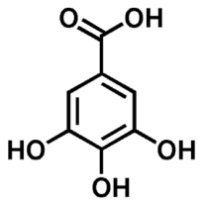	2.3	y = 28,245x + 478.4	0.9911
Catechin	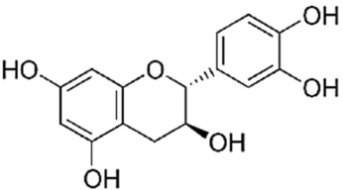	2.9	y = 21,015x + 8.6669	0.9972
Caffeic Acid	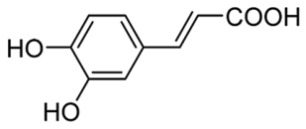	4.6	y = 42,330x − 157.55	0.9960
Sinapic Acid	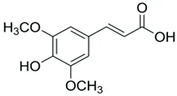	8.45	y = 38,244x − 85.881	0.9998
Kaempferol	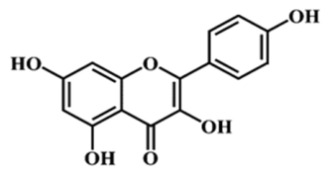	17.1	y = 202.47x − 2.1104	0.9966
Quercetin	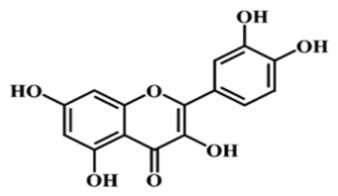	23.6	Y = 19,277x − 1233.2	0.9998

**Table 3:** Amount of polyphenols in samples, analyzed by the RP-HPLC method.

Sample	AM-L		AM-DP		AM-SOX	
Compound	Retention Time (min)	Concentration (mg/mL)	Retention Time (min)	Concentration (mg/mL)	Retention Time (min)	Concentration (mg/mL)
Gallic acid	2.2	1.32	2.2	0.26	2.2	0.78
Catechin	2.8	0.28	2.9	0.24	2.9	0.02
Caffeic acid	4.7	0.034	4.6	0.14	4.7	0.005
Sinapic Acid	8.4	0.11	8.6	0.68	8.6	0.036
Kaempferol	17.0	3.41	17.0	3.49	17.2	3.36
Quercetin	22.5	0.024	22.3	0.03	23.6	-

**Table 4:** Gravimetric results in 1 M HCl with increasing concentrations of *Achillea millefolium* extract.

HCl 1 M + AM Extract,	t = 48 h		t = 168 h		t = 216 h	
ppm GAE	P, mm/year	IE, %	P, mm/year	IE, %	P, mm/year	IE, %
0	7.6862	-	3.6872	-	1.7954	-
50	4.6324	39.73	1.8911	48.71	1.2494	30.41
100	3.4524	55.08	1.7090	53.65	1.2399	30.94
200	2.0575	73.23	1.6537	55.15	0.8867	50.61
300	1.8267	76.23	0.9894	73.17	0.4643	74.14

**Table 5:** Gravimetric results in NaCl 3.5% with increasing concentrations of *Achillea millefolium* extract.

NaCl 3.5% + AM Extract,	t = 48 h		t = 168 h		t = 216 h	
ppm GAE	P, mm/year	IE, %	P, mm/year	IE, %	P, mm/year	IE, %
0	0.0814	-	0.0342	-	0.0290	-
50	0.0484	40.46	0.0266	22.19	0.0266	8.42
100	0.0232	71.42	0.0186	45.53	0.0207	28.77
200	0.0266	67.34	0.0169	50.38	0.0181	37.70
300	0.0211	74.08	0.0119	65.26	0.0151	48.09

**Table 6:** Electrochemical parameters obtained from the Tafel analysis of carbon steel in 1 M HCl with the AM inhibitor.

1 M HCl + AM Extract, ppm GAE	E_cor_, mV	R_p_, Ω·cm^2^	i_cor_, mA/cm^2^	b_a_, mV/dec	−b_c_, mV/dec	P, mm/an	IE, %
0	−460.9	158.58	0.15	104.54	115.17	1.744	-
50	−417.1	394.02	0.0787	111.87	197.6	0.915	47.53
100	−405.3	449.89	0.0556	101.32	133.19	0.645	62.93
200	−417.9	557.3	0.0451	112.88	118.58	0.524	69.93
300	−411.03	538.47	0.0322	81.32	78.38	0.374	78.53

**Table 7:** Electrochemical parameters obtained from the Tafel analysis of carbon steel in NaCl 3.5% solution with the AM inhibitor.

3.5% NaCl + AM Extract, ppm GAE	E_cor_, mV	R_p_, Ω·cm^2^	i_cor_, mA/cm^2^	b_a_, mV/dec	−b_c_, mV/dec	P, mm/an	IE, %
0	−942.64	36.766	0.9426	176.76	145.47	10.953	-
50	−963.22	50.823	0.4799	115.32	109.46	5.576	49.08
100	−1009.1	60.299	0.4684	143.03	119.25	5.443	50.31
200	−975.88	57.44	0.4349	125.78	106.01	5.054	53.86
300	−969.75	82.147	0.1918	75.86	69.49	2.228	79.65

**Table 8:** Nyquist-derived electrochemical parameters for carbon steel in 1 M HCl with various AM extract concentrations.

1 M HCl + AM Extract, ppm GAE	R_s_, Ω·cm^2^	R_ct_, Ω·cm^2^	CPE1, Ω^−1^·cm^−2^·s^n n^		χ^2^·10^−3^	IE, %
			Y_0_·10^−4^	n		
0	2.061	156.00	3.83	0.78	4.28	-
50	1.509	317.27	2.76	0.78	3.26	50.83
100	2.051	327.95	2.40	0.78	4.97	52.43
200	3.982	423.02	2.29	0.76	3.35	63.12
300	14.561	504.69	1.60	0.80	2.85	69.08

**Table 9:** Nyquist-derived electrochemical parameters for carbon steel in NaCl 3.5% with various AM extract concentrations.

NaCl 3.5% + AM Extract, ppm GAE	R_S_, Ω·cm^2^	R_f_, Ω·cm^2^	CPEf, Ω^−1^·cm^−2^·s^n^		CPEdl, Ω^−1^·cm^−2^·s^n^		R_ct_, Ω·cm^2^	R_p_, Ω·cm^2^	χ^2^·10^−3^	IE, %
			Y_0_·10^−3^	n	Y_0_·10^−3^	n				
0	9.377	78.690	7.55	0.60	-	-	-	78.690	0.35	-
50	8.564	30.917	3.76	0.46	10.04	0.51	132.70	163.617	4.10	51.90
100	14.865	30.113	1.99	0.55	16.27	0.64	190.6	220.713	2.17	64.34
200	11.909	39.876	1.35	0.59	19.15	0.51	220.3	260.176	0.76	69.75
300	9.593	50.659	0.62	0.65	27.08	0.31	221.8	272.459	2.89	71.11

**Table 10:** Comparative data reports of some plant extracts used as green inhibitors.

Plant Extract	Major Phenolic Constituents	Metal/ Medium	Surface Analysis	IE%	Ref.
*Achillea millefolium*	gallic acid, catechin, caffeic acid, sinapic acid, kaempferol, quercetin	Fe/1 N HCl	-	78.53	This study
*Falcaria vulgaris*	genistin, rutin, quercetin, quercetin-3-O-glucozid	Fe/1 M HCl	AFM, SEM, XRD	91.30	[44]
*Galium verum*	gallic acid, catechin, vanillic acid, caffeic acid, kaempferol, quercetin, umbelliferon	Fe/1 N HCl	-	91.82	[15]
*Origanum majorana*	caffeic acid, quercetin	Fe/1 M HCl	SEM	93.06	[18]
*Achillea millefolium*	gallic acid, catechin, caffeic acid, sinapic acid, kaempferol, quercetin	Fe/NaCl 3.5%	-	79.65	This study
*Achillea millefolium*	phenols(unspecified)	Zn/rain water	-	23.40	[42]
*Catharanthus roseus*	quercetin, kaempferol, isorhamnetin	Fe/NaCl 3%	-	70.00	[9]
*Uncaria gambir*	tannin, catechin, flavonoid	Fe/NaCl 3.5% + 100 ppm NaHCO_3_	AFM, SEM, XRD	89.70	[43]

## Data Availability

The original contributions presented in this study are included in the article. Further inquiries can be directed to the corresponding authors.
